# The Role of Cytochrome c on Apoptosis Induced by *Anagrapha falcifera* Multiple Nuclear Polyhedrosis Virus in Insect *Spodoptera litura* Cells

**DOI:** 10.1371/journal.pone.0040877

**Published:** 2012-08-28

**Authors:** Kaiyu Liu, Duanyang Shu, Na Song, Zhongchao Gai, Yuan Yuan, Juan Li, Min Li, Shuying Guo, Jianxin Peng, Huazhu Hong

**Affiliations:** 1 Institute of Entomology, Central China Normal University, Wuhan, People's Republic of China; 2 Key Laboratory of Pesticide & Chemical Biology, Ministry of Education, Central China Normal University, Wuhan, People's Republic of China; Karolinska Institutet, Sweden

## Abstract

There are conflicting reports on the role of cytochrome c during insect apoptosis. Our previous studies have showed that cytochrome c released from the mitochondria was an early event by western blot analysis and caspase-3 activation was closely related to cytochrome c release during apoptosis induced by baculovirus in *Spodoptera litura* cells (Sl-1 cell line). In the present study, alteration in mitochondrial morphology was observed by transmission electron microscopy, and cytochrome c release from mitochondria in apoptotic Sl-1 cells induced with *Anagrapha falcifera* multiple nuclear polyhedrosis virus (AfMNPV) has further been confirmed by immunofluoresence staining protocol, suggesting that structural disruption of mitochondria and the release of cytochrome c are important events during Lepidoptera insect cell apoptosis. We also used Sl-1 cell-free extract system and the technique of RNA interference to further investigate the role of cytochrome c in apoptotic Sl-1 cells induced by AfMNPV. Caspase-3 activity in cell- free extracts supplemented with exogenous cytochrome c was determined and showed an increase with the extension of incubation time. DsRNA-mediated silencing of cytochrome c resulted in the inhibition of apoptosis and protected the cells from AfMNPV-induced cell death. Silencing of expression of cytochrome c had a remarkable effect on pro-caspase-3 and pro-caspase-9 activation and resulted in the reduction of caspase-3 and caspase-9 activity in Sl-1 cells undergoing apoptosis. Caspase-9 inhibitor could inhibit activation of pro-caspase-3, and the inhibition of the function of Apaf-1 with FSBA blocked apoptosis, hinting that Apaf-1 could be involved in Sl-1 cell apoptosis induced by AfMNPV. Taken together, these results strongly demonstrate that cytochrome c plays an important role in apoptotic signaling pathways in Lepidopteran insect cells.

## Introduction

Cytochrome *c* is an essential component of the mitochondrial respiratory chain. It is a soluble protein, localized in the intermembrane space, and is loosely attached to the surface of the inner mitochondrial membrane. The role of cytochrome c in mitochondria-mediated apoptosis signaling pathway of mammalian cell apoptosis has been thoroughly investigated [1]–[5]. Cytochrome *c* release from mitochondria is a key event and plays an important role in initiating apoptosis in the mammalian cells. Once released into the cytosol, cytochrome c binds to apoptotic protease-activating factor-1 (Apaf-1), leading to an unmasking of its caspase recruitment domain and the subsequent binding and autoproteolytic activation of procaspase-9. The complex of procaspase-9, cytochrome c and Apaf-1 are known as the apoptosome. Active caspase-9 then proteolytically activates downstream effector caspases, such as caspase 3, which degrades various cellular proteins propagating the apoptotic signal [5]–[11]. Because apoptosis is an evolutionarily conserved form and a common way for deleting unwanted and superfluous cells, it is considered that apoptosis regulation of insects has many parallels to vertebrate system.

However, the role of cytochrome c in insect cell apoptosis is not yet completely understood. There are the conflicting reports on cytochrome c release from the mitochondria to cytosol – important event in mitochondria-mediated apoptosis signaling pathway between the Diptera and Lepidoptera. In the *Drosophila* apoptotic system, cytochrome c is not involved in apoptosis in *Drosophila* cells such as S2 and BG2 cells [12]. Over-expression of cytochrome c in *Drosophila* BG2 cells or addition of recombinant cytochrome c to cytosolic BG2 extract did not lead to increased caspase activation or apoptosis, suggesting cytochrome c was not required for apoptosis in *Drosophila* BG2 cell lines [12]. Silencing of cytochrome c expression did not affect the induction of apoptosis in S2 cells [13]. The role of cytochrome c and other mitochondrial factors in caspase activation in *Drosophila* S2 cell extracts has been investigated, suggesting caspase activation in cultured *Drosophila* cell line S2 is regulated solely by cytoplasmic factors and does not involve any mitochondrial factors [14], [15]. The studies in *Drosophila* have demonstrated that cytochrome c is not released from mitochondria during apoptosis in the insect, and that cytochrome c plays no role in caspase activation in these cells. In contrast with reports in *Drosophila*, the release of cytochrome c from mitochondria and consequently activation of caspase-3 were demonstrated during lepidopteran cell apoptosis. The release of cytochrome c from mitochondria in ultraviolet-induced *Spodoptera frugipe*r*da* Sf-9 cells was observed by western blotting [16]. Antioxidants prevented UV-induced apoptosis by inhibiting mitochondrial cytochrome-c release and caspase activation in Sf-9 cells [17]. Cytochrome c added into the lysate of Sf-9 cells can also activate procaspase-3-like [18]. The study has shown that 2-deoxy-dribose is able to induce apoptosis, provoke cytochrome c release from the mitochondria and elicit caspase-3-like activity in *Lymantria dispar* IPLB-LdFB cells [19]. Cytochrome c release from the mitochondria in apoptotic *Spodoptera litura* cells induced with AfMNPV or ultraviolet has been detected by western blotting and cytochrome c may be required for caspase activation during the induction of apoptosis [2], [20]. Recent report indicated that cytochrome c release into the cytosol is an important event during lepidopteran Sf9 cells apoptosis induced with actinomycin D and may occur independent of mitochondrial membrane potential loss and MPTP (mitochondrial permeability transition pore) formation [21]–[23]. Caspase activation in Sf9 cell extracts is exclusively dependent on the release of cytochrome c [21]–[23].

In studies reporting Lepidopteran cell apoptosis, the release of cytochrome c during cell apoptosis was mainly based on western blot analysis. Here, we used morphology observation, co-localization analysis, cell-free extract system and the technique of RNA interference to investigate the role of cytochrome c in apoptotic Sl-1 cells induced by AfMNPV.

## Results

### Mitochondrial morphological changes in cells undergoing apoptosis

**Figure 1:**
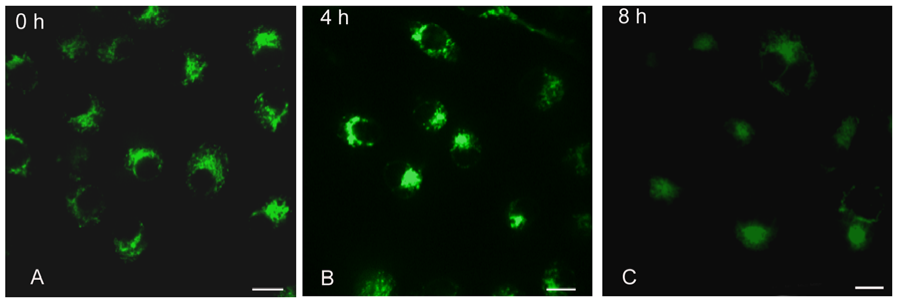
Changes of mitochondria in apoptotic cells induced with AfMNPV under confocal microscopy. (A) Control cells stained with Mito-Tracker Green, showing mitochondrial normal distribution; (B) Infection of AfMNPV caused mitochondria to aggregate at 4 h post-infection; (C) Mito-Tracker Green staining showed a diffusion of fluorescence at 8 h post-infection, suggesting the disruption and depolarization of mitochondria. Bar = 10 **µ**m.

Mito-Tracker Green staining showed that mitochondria disturbed in cytoplasm in normal Sl-1 cells (Fig. 1A). However, the infection of AfMNPV caused mitochondria to aggregate at 4 h post-infection (Fig. 1B). At 8 h of post-infection, Mito-Tracker green staining showed a diffusion of fluorescence, suggesting the disruption and depolarization of mitochondria (Fig. 1C).

**Figure 2:**
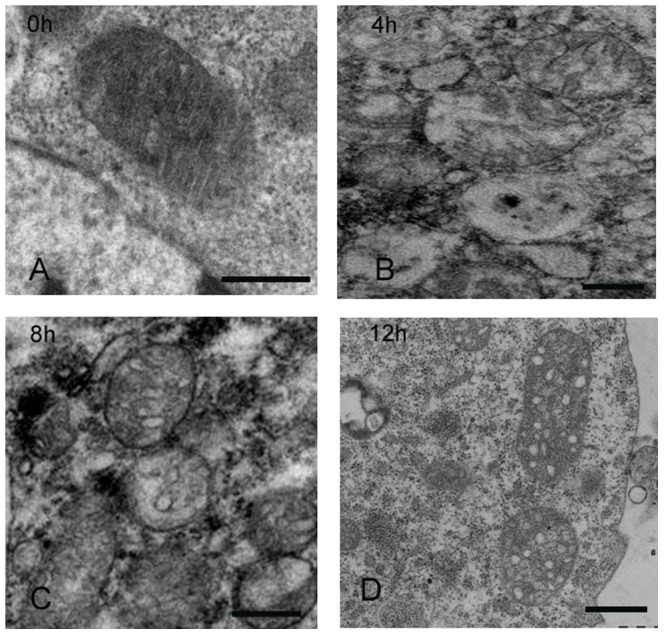
Transmission electron images of mitochondria in apoptotic cells induced with AfMNPV. (A) Control cells showing mitochondria had normal ultrastructure with intact cristae and an elongated form; (B) AfMNPV infection for 4 h resulted in change of mitochondria that appeared swollen and rounded; (C) Mitochondria were further swollen and rounded, cristae of which disappeared in cells infected by AfMNPV for 8 h; (D) Mitochondria with swollen and vesicular-swollen morphology in cells infected with AfMNPV for 12 h. Bar = 500 nm.

The ultrastructure of mitochondria was observed to determine whether morphological changes of mitochondria occurred in cells undergoing apoptosis induced with AfMNPV. Virus infection resulted in a very obvious alteration in mitochondrial morphology (Fig. 2). In normal cells, mitochondria are in an elongated form with intact cristae (Fig. 2A). In cells infected with AfMNPV, these cells displayed abnormal mitochondrial morphology. The electron micrographs showed that some mitochondria appeared swollen and rounded at 4 h post-infection (Fig. 2B). Mitochondria were further swollen and cristae of mitochondria disappeared in cells at 8 h post –infection (Fig. 2C). Mitochondria showed swollen and vesicular-swollen morphology at 12 h post-infection (Fig. 2D).

These observations demonstrated that mitochondria were disrupted in apoptotic SL-1 cells induced by AfMNPV. Similar to mammalian cell apoptosis, the morphological changes in mitochondria was also an important feature in insect apoptotic cells.

### Cytochrome c co-localization in Sl-1 cells undergoing apoptosis

**Figure 3:**
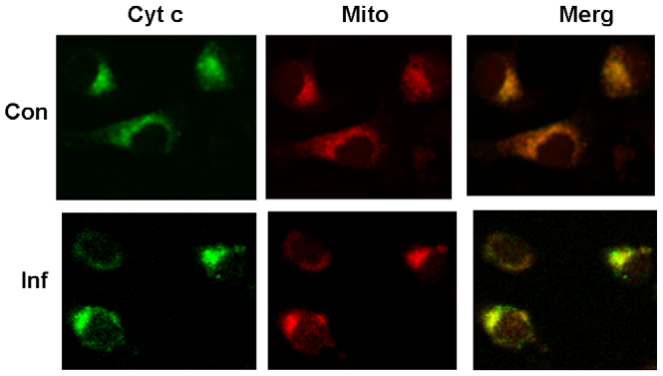
Cytochrome c localization in Sl-1 cells was altered during apoptosis. Sl-1 cells infected with AfMNPV for 6 h were co-stained with anti -cytochrome c monoclonal antibody and Mito-Tracker red. After incubation with FITC-coupled secondary antibodies, cells were visualized by confocal laser scanning microscopy. Con: Control cells treated without AfMNPV; Inf: Cells infected with AfMNPV for 6 h.

To determine cytochrome c localization during apoptosis, Sl-1 cells were stained by double fluorescence staining. In control cells, the co-localization of cytochrome c and mitochondria was observed by both Mito Tracker red and coupled –FITC cytochrome c antibody. In cells undergoing apoptosis, cytochrome c did not colocalized with the mitochondria (Fig. 3), suggesting cytochrome c localization was altered in Sl-1 cells undergoing apoptosis induced by AfMNPV.

### Cytochrome c induces caspase -3 activation in Sl-1 cell-free extracts

**Figure 4:**
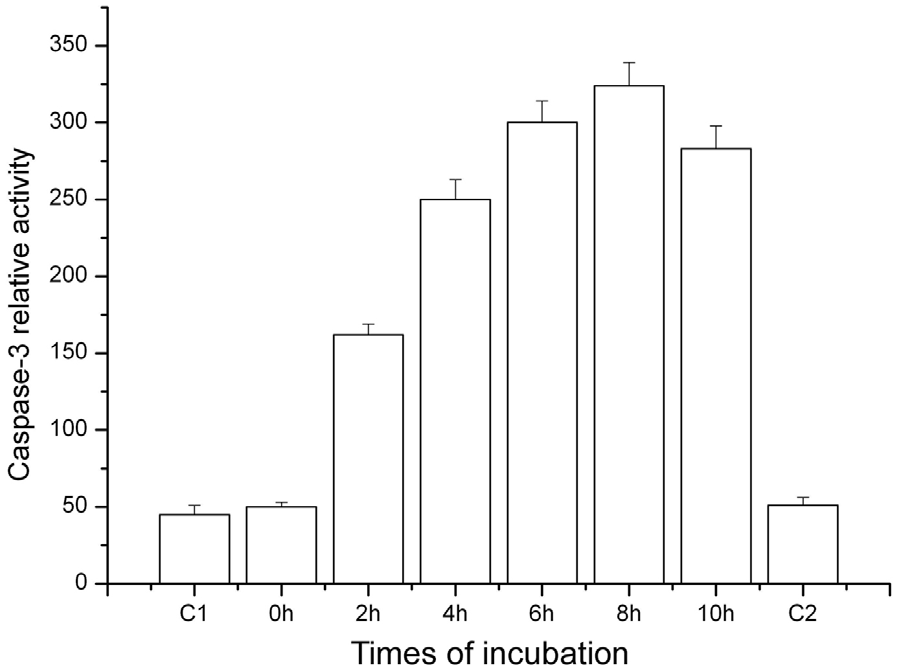
Cytochrome c stimulated caspase-3 activity in cell-free extracts. Caspase-3 activity in the presence of cytochrome c was measured by a flourimetric assay using the substrate Ac-DEVD-AFC. C1: Control untreated with cytochrome c; 0–10 h: caspase-3 activity in samples treated with cytochrome c for different time points; C2: control treated without cytochrome c for 8 h.

Cytochrome c is critical for caspase activation and the resulting consequence of the cytochrome c release generally gives rise to caspase activation in mammalian cells. To test whether cytochrome c can induce caspase activation in a cell-free extracts, cytochrome c was added to Sl-1 cell extracts and caspase-3 activation was tested by a fluorescent assay using the substrate Ac-DEVD-AFC. It was found that the addition of cytochrome c increased caspase-3 activity. Caspase-3 activity evidently increased with incubation time extension in the presence of cytochrome c (Fig. 4). Under similar conditions, no increasing of caspase-3 activity was measured in the absence of cytochrome c. These results revealed that cytochrome c could induce and promote caspase-3 activation in Sl-1 cell-free extracts. It is suggested that the signal for caspase-3 activation is directly related to cytochrome c.

### DsRNA silences cytochrome c expression in Sl-1 cells

**Figure 5:**
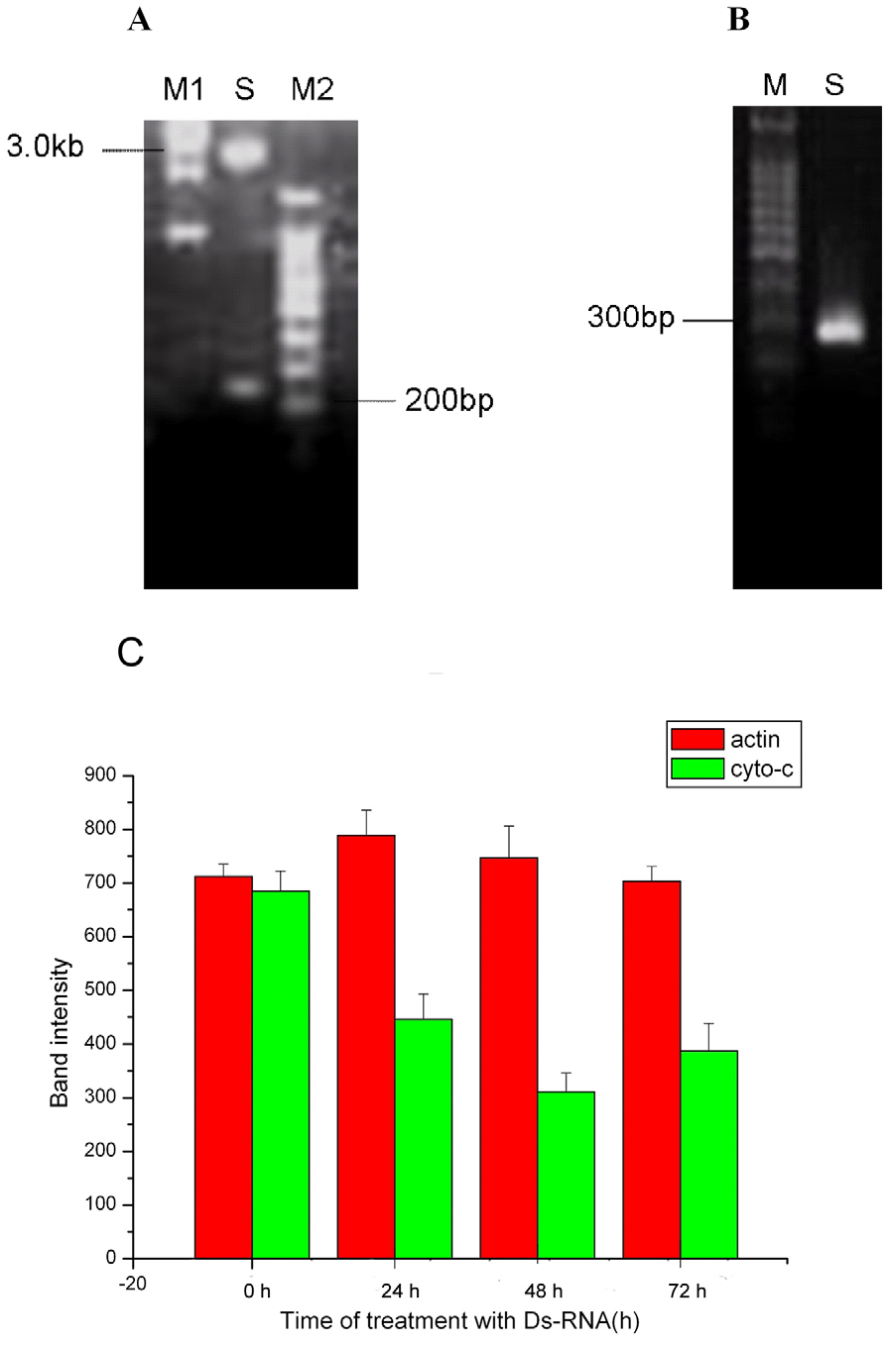
Cytochrome c dsRNA silenced cytochrome c expression in Sl-1 cells. (A) Identification of recombinant plasmid pLitmus-cytc containing cytochrome c DNA fragment. pLitmus-cytc was digested with BamH I and EcoR I and then analyzed by agarose gel electrophoresis. M1: 1 kb maker, M2: 100 bp maker, S: plasmid DNA products. (B) Electrophoretic analysis of dsRNA transcribed *in vitro*. pLitmus-cyt c was digested with BamH I and EcoR I respectively and transcribed *in vitro*, and RNA products were electrophorised on agrose gel. M: 100 bp maker, S: RNA transcription products. (C) Cytochrome c mRNA level after treatment with dsRNA. Sl-1 cells treated with dsRNA for 0, 24, 48, and 72 h, and after inoculated with AfMNPV for 10 h, total RNA in each treatment was isolated. Cytochrome c mRNA was determined by semi-quantitative RT-PCR.

Based on RT-PCR amplification, sequence alignment and sequencing, the recombinant plasmid pLitmus-cyt c which contained partial sequence of *Spodoptera litura* cell cytochrome c gene and the T7 promoter sequence on both ends of the insert had been constructed and was identified by electrophoresis (Fig. 5A). DsRNAs corresponding to the coding regions of cytochrome c were transcribed *in Vitro* and the transcription productions were identified by electrophoresis (Fig. 5B). To investigate the effect of dsRNA on cytochrome c expression, semi-quantitative RT-PCR was used to verify cytochrome c expression levels. Sl-1 cells were treated with dsRNA at different time points and total RNA was isolated. Cytochrome c mRNA levels were determined with semi-quantitative RT-PCR. Levels were relative to mRNA expression in cells treated with GFP dsRNA and normalized with an internal control actin gene. Results showed that cytochrome c mRNA levels decreased and was down-regulated after transfection with cytochrome c dsRNA (Fig. 5C).

**Figure 6:**
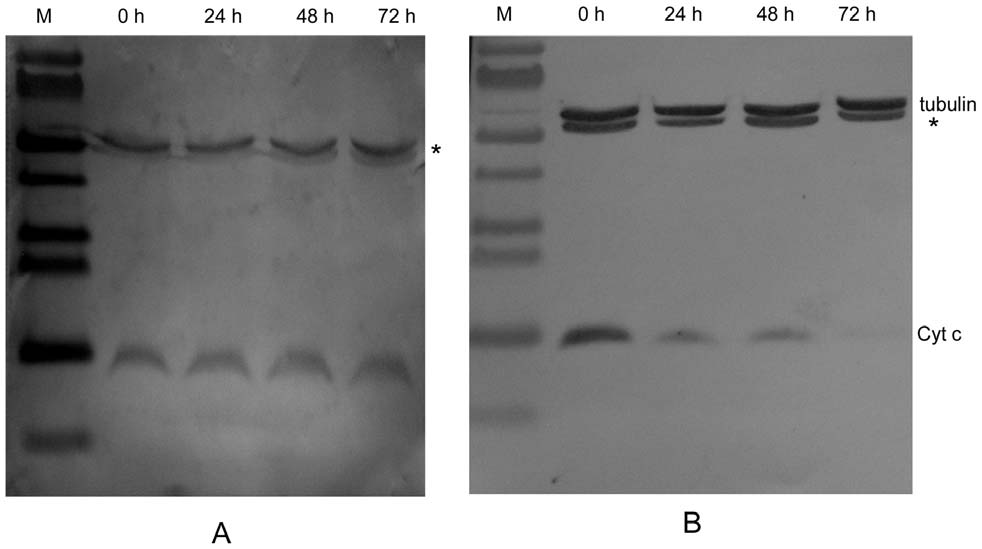
Western blot analysis showed the decrease of abundance of cytochrome c after transfection of its dsRNA in Sl-1 cells. (A) Sl-1 cells transfected with GFP dsRNA; (B) Sl-1 cells transfected with cyt-c dsRNA. M, protein marker. β-tubulin was used as an internal control. *: non-specific band.

Western-blot was further used to confirm the down-regulation of cytochrome c after transfection with cytochrome c dsRNA. The total protein of cells were extracted at different time points after transfection and determined according the methods of BCA. β-tubulin was used as an internal control to normalize the relative level of cytochrome c. The results demonstrated that the abundance of cytochrome c decreased gradually during 72 h of post-transfection with cytochrome c dsRNA (Fig. 6).

### Silencing of cytochrome c prevents AfMNPV –induced cell apoptosis

**Figure 7:**
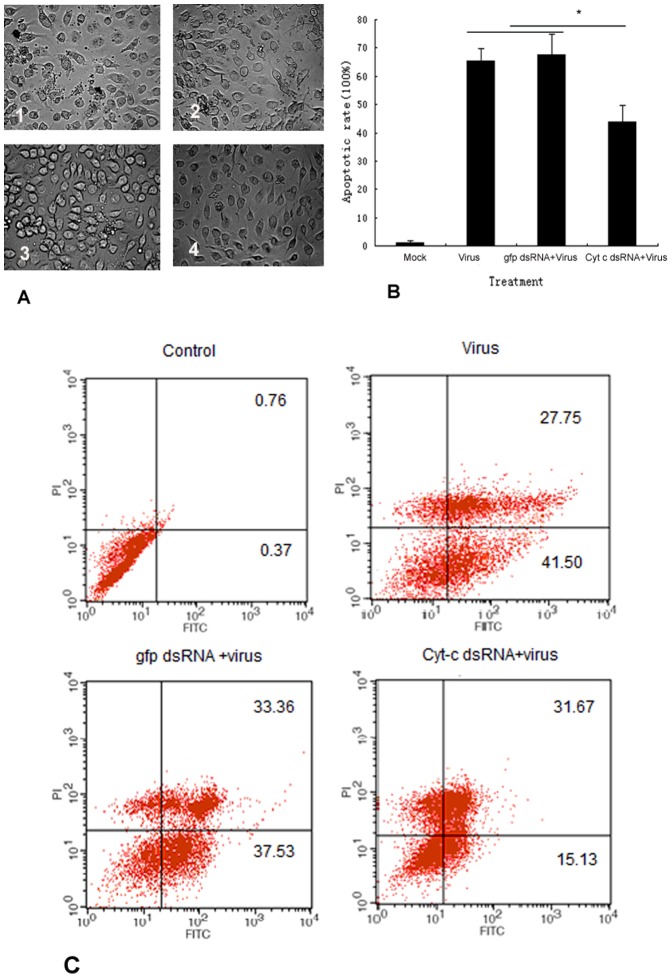
Effect of silencing of cytochrome c on apoptosis induced by AfMNPV in Sl-1 cells. (A) Microscopy image of cells treated with AfMNPV and cyt-c dsRNA. 1. Cells infected with AfMNPV alone for 10 h; 2. Cells infected with AfMNPV for 10 h after treatment with cyt c dsRNA for 24 h; 3. Cells infected with AfMNPV for 10 h after treatment with cyt-c dsRNA for 48 h.; 4. Cells infected with AfMNPV for 10 h after treatment with cyt-c dsRNA for 72 h. (B) Flow cytometric analysis. Sl-1 cells treated with dsRNA for 48 h,and infected with AfMNPV. At 10 h post infection, cells were staining by PI and FITC-Annexin. Apoptosis was analyzed by flow cytometry. (C) Cyt-c dsRNA resulted in the decrease of apoptosis induced by AfMNPV in Sl-1cells. Data were representive for three independent experiments. *, *p*<0.05.

To determine whether silencing of cytochrome c expression affected apoptosis in Sl-1 cells induced with AfMNPV, we treated cells with double–stranded RNA transcribed from cytochrome c cDNA at different time points. Cells treated with dsRNA had been infected by AfMNPV for 10 h and then visualized. Morphological observation showed that a large number of apoptotic bodies appeared in cells infected with the virus (Fig. 7A1). After treatment with cytochrome c dsRNA, the number of apoptotic cells induced with AfMNPV declined significantly, and apoptotic bodies decreased gradually with time extension after cytochrome c dsRNA treatment (Fig. 7 A2, 3 and 4). Morphological feature showed that cytochrome c dsRNA interfered with the induction of apoptosis with the virus in Sl-1 cells. To detect quantitatively apoptotic cells, cells transfected with dsRNA at 48 h were analyzed by flow cytometry. The flow cytometry analysis showed that cytochrome c dsRNA caused the decline of the apoptotic rate in AfMNPV-induced Sl-1 cells (Fig. 7A and B). These results indicated that cytochrome c dsRNA treatments resulted in a significant reduction of apoptotic rate. Apparently, silencing of cytochrome c inhibited apoptosis induced by AfMNPV in Sl-1 cells.

### Silencing of cytochrome c inhibited the activation of pro-caspase 3 and pro-caspase 9 in SL-1 cells induced by AfMNPV

**Figure 8:**
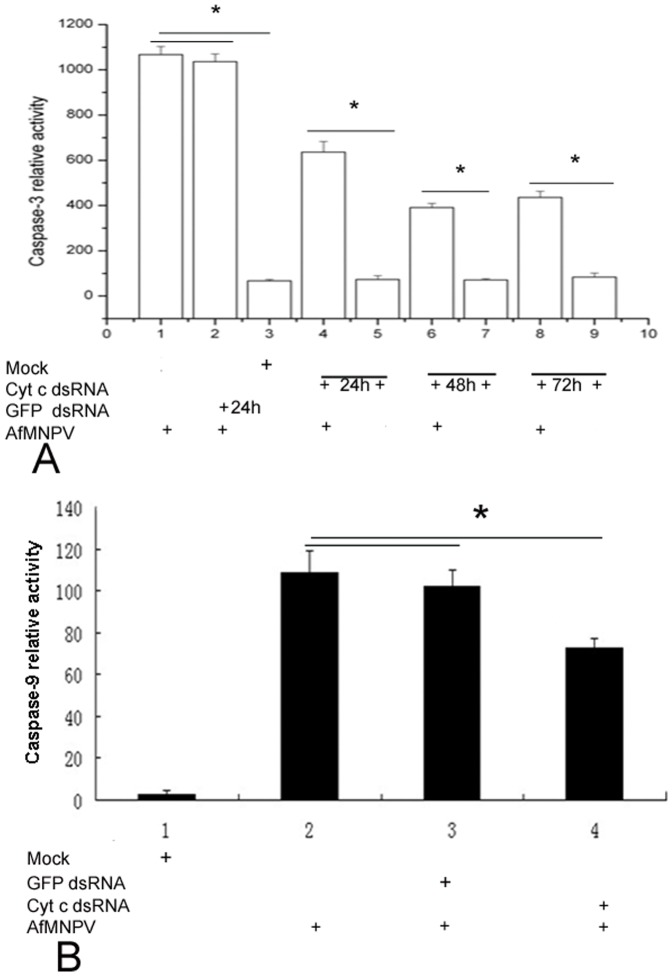
Down-regulation of cytochrome c resulted in the reduction of caspase 3 and caspase-9 activity in AfMNPV-induced Sl-1 cell. (A) Sl-1 cells treated with dsRNA for different time points, apoptosis was induced with AfMNPV, and then caspase-3 activity was measured at 10 h post-infection.1. Control cells treated alone with AfMNPV; 2. Cells treated with GFP dsRNA and virus; 3. Cells without any treatment; 4. Cells treated with dsRNA, 24 h later, infected by AfMNPV for 10 h; 5. Cells treated alone with dsRNA for 24 h; 6. Cells treated with dsRNA, 48 h later, infected by AfMNPV for 10 h; 7. Cells treated alone with dsRNA for 48 h; 8. Cells treated with dsRNA, 72 h later, infected by AfMNPV for 10 h; 9. Cells treated alone with dsRNA for 72 h. (B) Caspase-9 activity in cytochrome c dsRNA-treated Sl-1 cells after infection with AfMNPV for 10 h, compared with control cells.1. Normal cells; 2. Cells infected with AfMNPV; 3. Cells infected with AfMNPV for 10 h after GFP dsRNA treatment for 48 h; 4. Cells infected with AfMNPV for 10 h after cyt c dsRNA treatment for 48 hr. *, *p*<0.05.

To examine the effect of the expression knockdown of cytochrome c on caspase-3 activation in Sl-1 cells, we conducted cytochrome c dsRNA inference as well as caspase-3 activity assays. Cells were treated with dsRNAs corresponding to the coding region of cytochrome c for 24, 48 and 72 h, followed by infection with AfMNPV at 5 moi for 10 h, cytosolic fractions from Sl-1 cells undergoing apoptosis were used as caspase-3 activation assay. The caspase-3 activity assay performed for the cells incubated in the presence of cytochrome c dsRNA and AfMNPV revealed a significant decrease in caspase-3 activity with respect to the control cells treated with GFP dsRNA and without cytochrome c dsRNA and other treatments. At 24 h post treatment of cytochrome c dsRNA, cells infected with the virus showed that caspase-3 activity decreased significantly. At 72 h after incubation, the caspase-3 activity still was markedly lower than the control cells after various treatments (Fig. 8A).

To determine whether the decreased levels of cytochrome c expression would also affect caspase-9 activity in Sl-1 cells, we measured caspase-9 activity in cells infected with AfMNPV in the presence of dsRNA. The result showed that interfering cytochrome c expression caused the reduction of the level of caspase-9 activity (Fig. 8B).

Silencing of cytochrome c expression remarkably influenced the caspase-3 and caspase-9 activity in apoptotic Sl-1 cells infected with AfMNPV, suggesting that the caspase-3 and caspase-9 activity in Sl-1 cells is closely associated with cytochrome c.

### Caspase-9 was involved in the activation of pro-caspase-3 in Sl-1 cells infected with AfMNPV

**Figure 9:**
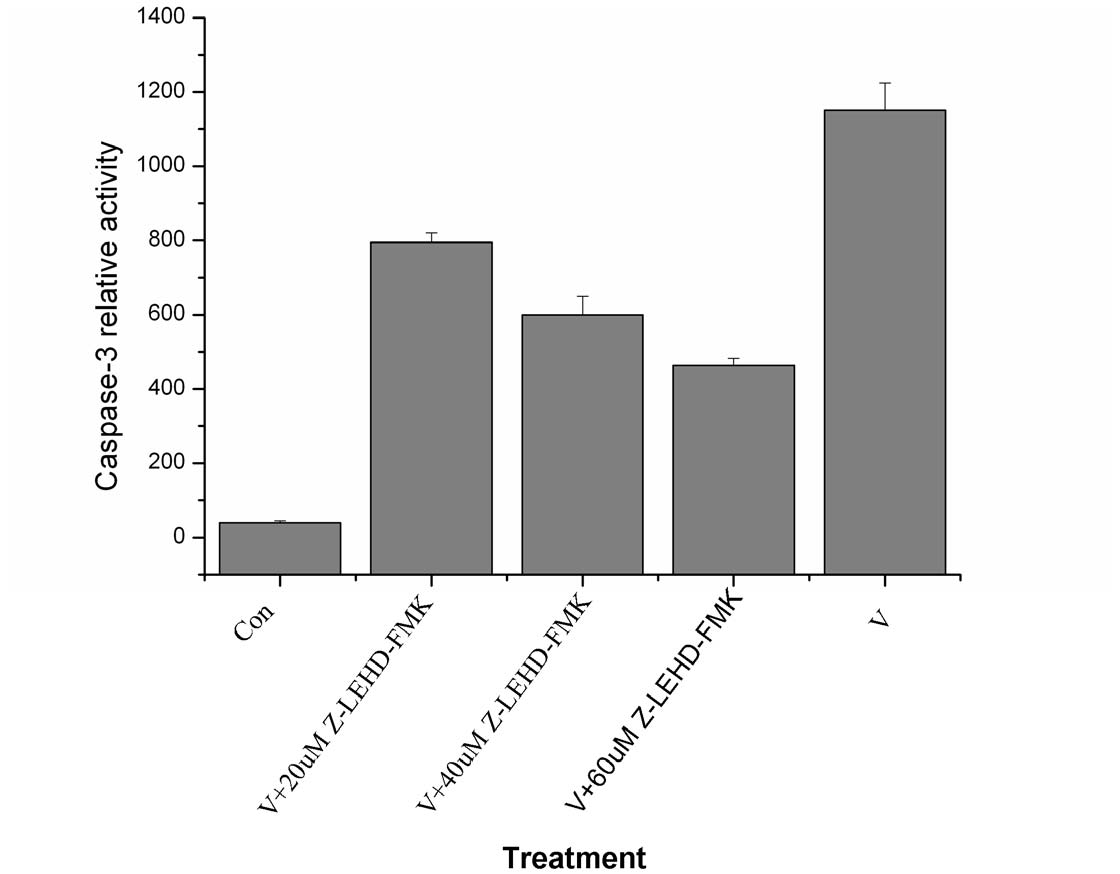
Inhibitor of caspase-9 blocked the activation of pro-caspase-3 in AfMNPV-induced Sl-1 cells. Con: non-infected cells; V: AfMNPV-infected cells; V+Z-LEHD-FMK: AfMNPV-infected cells cultured in complete medium supplemented with inhibitor of caspase-9 (Z-LEHD-FMK).

In order to determine the relationship between caspase-9 and caspase-3 cascade, Sl-1 cells were infected by AfMNPV in the presence or absence of caspase-9 inhibitor (Z-LEHD-FMK) and caspase-3 activities were measured by a spectrofluoremeter at 10 h post-infection. The results demonstrated that the inhibition of caspase-9 activity significantly blocked the activation of pro-caspase-3 and the effect was in a dosage-dependent manner (Fig. 9), suggesting the activation of procaspase-9 was the upstream event during the process of apoptosis in Sl-1 cells induced with AfMNPV.

### Inhibition of the function of Apaf-1 blocked apoptosis

**Figure 10:**
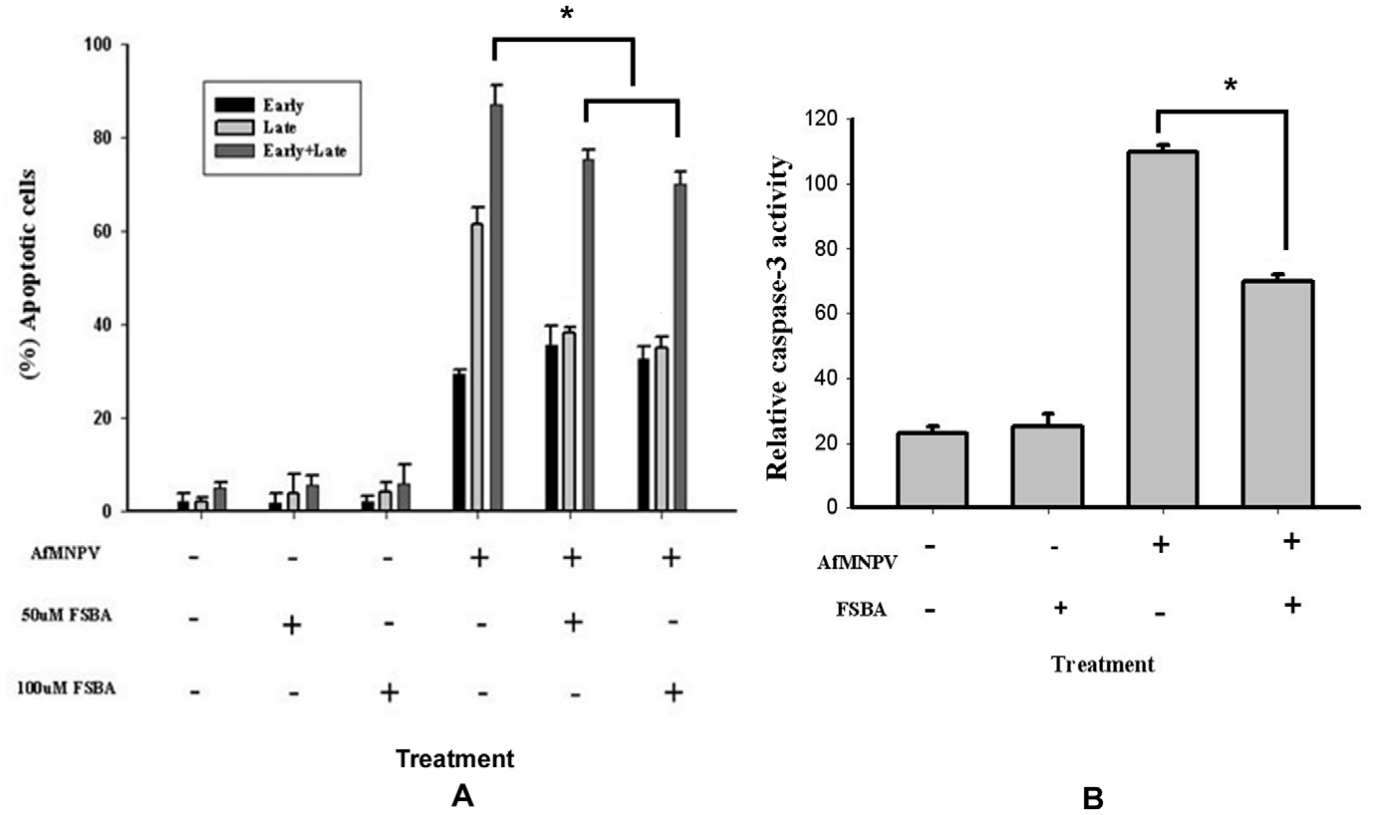
Inhibition of the function of Apaf-1 blocked apoptosis. (A) The treatment of FSBA resulted in the decrease of the percentage of apoptotic cells under the inductuion of AfMNPV. (B) The treatment of FSBA inhibited activation of pro-caspase-3 in AfMNPV-infected Sl-1 cells. *, There were significant differences.

5′-p-fluorosulfonylbenzoyl adenosine (FSBA), an ATPase inhibitor, is known to inhibit the function of Apaf-1/CED-4-like molecules and could result in the blockage of the caspase activation in the embryo of Drosophila and Sf9 cells of *Spodoptera frugiperda* [24], [25]. To determine the function of *S. litura* Apaf-1 in the process of insect cell apoptosis, the relative caspase-3 activity and the percentage of cell apoptosis were measured as described above after Sl-1 cells were treated with FBSA and the inducers of apoptosis (AfMNPV). The results demonstrated that FBSA could inhibit the AfMNPV-induced apoptosis in Sl-1 cells (Fig. 10). In addition, FBSA could also inhibit the actinomycin D-induced apoptosis in Sl-1 cells (data not shown). These results strongly suggest that Apaf-1 plays an important role during apoptosis of Sl-1 cells, which was known to involve in the cytochrome c-induced apoptosome formation.

## Discussion

Cytochrome c has a key role in the mitochondrial–mediated apoptosis pathway and triggers caspase activation as well as caspase cascade in mammalian cells [8], [26], [27]. Our previous work has demonstrated the release of cytochrome c from mitochondria into cytosol and subsequent activation of caspase-3 in *S. litura* cells (Sl-1 cell line) undergoing apoptosis induced by various stimuli, such as baculovirus or UV, but these results were primarily based on western blot analysis [2], [20]. In this paper, we further demonstrated the release of cytochrome c from mitochondria into the cytosol in apoptotic Sl-1 cells by using immunofluoresence staining. Meanwhile, electron microscopy revealed that mitochondria were disrupted in SL-1 cells undergoing apoptosis induced with AfMNPV. In *Drosophila*, expression of Hid or Rpr resulted in mitochondrial disruption [28], which indicates the morphological change of mitochondria is a common feature of apoptotic insect cells. Our data suggests that the release of cytochrome c could be associated with the alteration of mitochondria morphology in *Lepidopteran* cell apoptosis. Because the role of cytochrome c on apoptosis signaling is an important and controversial issue regarding insect cell apoptosis, we used the technique of RNA interference and a cell-free extract system to further confirm the role of cytochrome c on apoptosis induced by AfMNPV in *Lepidopteran* Sl-1 cells. The present study showed the addition of exogenous cytochrome c to Sl-1 cell-free extracts was sufficient to induce caspase-3 activation which indicates that cytochrome c is required for caspase-3 activation in *Lepidopteran* Sl-1 cells. More recent studies showed the addition of purified cytochrome c or mitochondria lysates to Sf9 cell-free extracts induced DEVDase activity [21]. When cytochrome c was deleted with anti-cytochrome c antibody, DEVDase had been not observed [21]. The role of cytochrome c in both Sl-1 cell-free and Sf9 cell-free extracts is consistent and has parallel to mammalian cell-free extracts. In *Drosophila*, one study had shown that cytochrome c induced a small amount of caspase activation in embryo extracts but there was a 2.5-fold induction of caspase activity, which contrasts with the 50–100-fold induction or more in vertebrate cell extracts in that study [29]. However, the two cytochrome c species, DC3 and DC4, failed to induce caspase activation and promote caspase activation in *Drosophila* cell extracts, but remarkably induced caspase activation in extracts from human cells [30], [12]. There is distinctive difference on the role of cytochrome c in both *Lepidopteran* cell-free and *Drosophila* cell-free systems.

The dsRNA-mediated gene silencing has been used to silence the expression of targeted genes associated in apoptosis, such as cytochrome c, Apaf-1-related killer (ARK, apoptosis-related killer) in *Drosophila* cells [12], [13]. In order to facilitate the functional study of cytochrome c on apoptosis in insect cells, we silenced the expression of cytochrome c by RNA interference in *Spodoptera litura* cells with dsRNAs to examine the effects of silencing of cytochrome c on apoptosis induced with AfMNPV. Silencing of cytochrome c expression in Sl-1 cells resulted in the inhibition of apoptosis induced with AfMNPV. Once dsRNA was introduced into the cells, the apoptotic rate significantly decreased, indicating silencing of cytochrome c protected the cells from the death infected by AfMNPV. The mechanism underlying the protective effect of silencing cytochrome c appears to involve an activation of caspase or change of caspase activity. Therefore, we also set out whether silencing of cytochrome c affected caspase-3 and caspase-9 activity in Sl-1 cells. Knockdown of cytochrome c led to a significant decrease of caspase-3 and caspase-9 activity suggesting the activity of caspase-3 and caspase-9 activity is dependent upon cytochrome c in *Lepidopteran* cells.

For the studies reported in *Drosophila*, silencing both dc3 and dc4 in BG2 cells had no effect on the processing of Dronc (a homologue of mammalian caspase-9) and Drice (a homologue of mammalian caspase-3) or caspase activity after induction of apoptosis by cyloheximide, indicating cytochrome c is not required for caspase activation in *Drosophila* cells [12]. Although *Drosophila* cytochrome c protein may be not required for apoptosis and caspase activation, accumulating evidence has shown that cytochrome c involved in apoptosis and caspase activation in Lepidopteran insect apoptosis [2], [16], [17], [19], [20], [21]. It is considered that the molecular mechanisms of apoptosis are highly conserved throughout evolution. Why does a distinctive difference exist on the role of cytochrome c in apoptosis in Lepidopteran and Diptera insects? The report pointed that the findings in *Drosophila* are based largely, if not exclusively, on studies in cultured S2 cells which are phagocytic cells derived from embryonic macrophages/hemocytes, suggesting that macrophages are uniquely resistant to apoptosis among embryonic cell types, and that S2 cell line is not a suitable general model for *Drosophila* cell death [31]. We do not know whether the differences of the cytochrome c role between two kinds of insects during apoptosis are due to different cell types. Perhaps this difference reflects the differences of the evolution between Lepidopteran and Diptera insects. Therefore, the role of cytochrome c during insect cell apoptosis should be investigated in more insect cell lines or tissue types.

Cytochrome c plays an important role for apoptotic signaling and may be required for Lepidopteran insect apoptosis. We speculate that the role of cytochrome c in Lepidopteran cells and mammalian cells apoptosis is homologous. The function of cytochrome c in mammalian cell apoptosis involves APaf-1 activation and apoptosome formation. We have obtained the EST of *Slapaf-1* (Unpublished) in Sl-1 cells, which shares high homology with *Bombyx mori Bmapaf-1* [32] and *Manduca sexta Msapaf-1*(Unpublished), and the elucidation of the apoptosome formation is undergoing in Lepidopteran cells.

**Figure 11:**
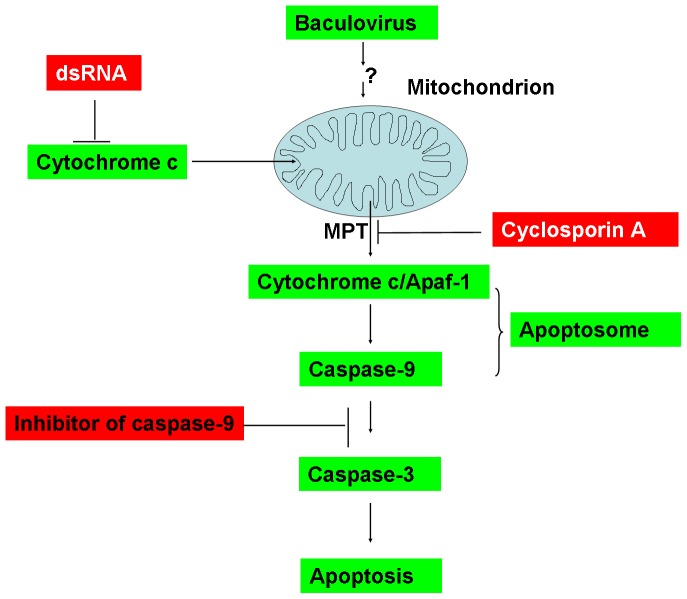
Pathway of apoptosis mediated by cytochrome c in AfMNPV-infected Sl-1 cells. MPT: mitochondrial permeability transition.

In summary, based on the present studies and the previous reports [2], [17], [20], [21], [22], [33], [34], the pathway of apoptosis mediated by cytochrome c has been given a clue in AfMNPV-infected Sl-1 cells. In the present studies, we have provided further evidence that cytochrome c is essential for AfMNPV- or actinomycin D-induced apoptosis through cytochrome c-induced apoptosome formation (Fig. 11).

## Materials and Methods

### Cell and virus

*Spodoptera litura* cell line (SL-ZSU-1 or Sl-1) was obtained from the Institute of Entomology, Zhongshan University [35]. *Anagrapha falcifera* multiple nuclear polyhedrosis virus (AfMNPV) was a gift of Dr. McIntoch in Department of Agriculture, USA [36].

### Cell culture and induction of apoptosis

SL-1 cells were propagated in GIBCO™ Grace's medium (Invitrogen, Grand Island, N.Y., USA) supplemented with 10% fetal bovine serum (FBS) (Invitrogen, Grand Island, N.Y., USA), 0.3% yeast extract and 0.3% lactalbumin hydrolysate at 28°C. For induction of apoptosis, 5×10^5^ cells/ml were seeded into flasks or 6-well plates, after cells attached the bottom of the plate, the medium was removed and cells were inoculated with AfMNPV at multiplicity of infection (MOI) of 5 for 1.5 hr. The virus was removed and cells were washed twice with serum-free medium and then cultured in Grace's medium supplemented with 10% FBS. The morphological changes of apoptotic cells were visualized with microscopy.

### Observation of mitochondrial morphology

Sl-1 cells were grown on coverslips (20 mm×20 mm) in 6-well plates overnight. After infection with AfMNPV for different time periods, cells were incubated with 300 nM Mito-Tracker Green (Molecular Probes Company) for 15 min at room temperature. After the stained cells were washed twice with PBS, they were visualized under confocal microscopy according to the instruction provided by the manufacturer.

Sl-1 cells cultured in flasks were washed tree times in PBS buffer after cells were infected with AfMNPV at 0 h, 4, 8 and 12 h post infection respectively and then cells were pre-fixed in 2.5% glutaraldehyde for 30 min and fixed in 2.5% glutaraldehyde for 2 h at 4°C. The specimens were conventionally dehydrated, infiltrated, embed and sectioned. Thin sections were stained with uranyl acetate and observed under a transmission electronic microscopy (Philips 2 Tecnai 10).

### Immunofluoresence staining

Cells were grown on coverslips (10 mm×10 mm) in 24-well plates. After apoptosis induced with AfMNPV, cells were washed twice with PBS and incubated with 300 nM Mito-Tracker Red CMXRos in PBS with 0.8% BSA for 15 min at room temperature. Cells were washed twice with PBS and fixed with 4% cold paraformaldehyde for 10 min at room temperature. The washed cells were permeabilized with 0.1% Triton X-100 for 5 min at room temperature and washed three time with PBS and then were blocked with 1% BSA for 30 min at room temperature. Cells were incubated with anti-cytochrome c mouse monoclonal antibody (BD Biosciences) overnight at 4°C. After cells were washed three times in PBS, cells were stained with FITC-conjugated secondary antibody for 90 min at room temperature and then washed three times with PBS. Coverslips were mounted onto slides and observed with confocal laser scanning microscopy at an excitation wavelength of 488 nm and an emission wavelength of 515 nm for FITC, and at 595 nm excitation wavelength and 570 nm emission wavelength for Mito-Tracker Red CMXRos.

### Preparation of cell-free extracts

SL-1 cells were harvested and washed twice with ice-cold PBS. The cells were suspended in 5 ml lysis buffer (20 mM Hepes -KOH, pH 7.5, 10 mM KCl, 1.5 mM MgCl_2_, 1 mM EDTA-Na_2_, 1 mM EGTA-Na_2_, 1 mM DTT, 250 mM sucrose), supplemented with protease inhibitors (0.1 mM PMS, 6 mg/ml aprotinin, 8 mg/ml leupeptinin and 10 mg/ml pepstain). After sitting on ice for 10 min, the cells were homogenized with a glass homogenizer. The homogenates were centrifuged at 100,000 g for 1 h at 4°C. The supernatant was further centrifuged at 100, 000 g for 30 min. The resulting supernatants were saved as cell-free system and stored at −80°C in multiple aliquots for the *in vitro* apoptosis assay.

### Apoptosis assay in cell-free system

Cytochrome c was added into the cell-free system at a final concentration of 5 µmol/l and incubated at 28°C. The cell-free system treated with cytochrome c was designated as the apoptotic reaction system. The reactive system treated without cytochrome c was used as the control. Apoptosis in the cell-free system was determined and confirmed by the caspase-3 activity assay.

### Construction of recombinant plasmid containing cytochrome c DNA fragment

RNA was isolated from Sl-1 cells (1.5×10^6^ cells) with Trizol (GIBCO-BRL) extraction according to manufacturer's instruction. cDNA was generated using 1 µg total RNA and 1 µl M-MLV reverse transcriptase (3 U/µl) (Promega). Based on the sequence homology from Lepidopterans such as *Bombyx mori*, *Trichoplusia ni*, *Manduca sexta* cytochrome c genes, A pairs of degenerate primers for cytochrome c (forward primer, 5′-GGGTGTWCCTGCWGGMAATGCT-3′; reverse primer, 5′-GRTCTGCACGYTCRTTKGCCT-3′.) was designed for PCR. Total volume of 50 µl PCR reaction system consisted of 5 µl 10×PCR buffer,1 µl cDNA template, 1 µl forward primer, 1 µl reward primer, 0.5 µl Taq DNA polymarase(4 U/µl), 2 µl 10 mM dNTP and ddH_2_O. The PCR product was electrophorised on 1.5% agarose gel and recovered from gel by Axygen kit according to protocol outlined by manufacturer. The purified DNA was cloned into T-vector and sequenced. The DNA sequences with 293 bp were analyzed with the cytochrome c genes from *Bombyx mori* and *Trichoplusia ni* with NCBI-Blast software and the identity of *Spodoptera litura* cytochrome c DNA fragments compared with silkworm and cabbage lopper cytochrome c genes were 83% and 84% respectively. According the sequence of *Spodoptera litura* cytochrome c DNA fragment, the sequence specific primer containing BamH I and EcoR I sites for cytochrome c, forward primer, 5′-CGGGATCCGATCTGCACGCTCGTTTGCCT-3′ (underline, BamH I site) and reverse primer, 5′-CGGAATTCTGCCACACTGTTGAAGCCGGT-3′ (underline, EcoR I site) were redesigned. *Spodoptera litura* cytochrome c DNA fragments (248 bp) were amplified by PCR and analyzed by 1.5% agarose gel electrophoresis and recovered. The plasmid pLitmus28i (New England BioLabs., Inc.) DNA was extracted and then plasmid DNA and cytochrome c DNA fragments were digested with BamH I and EcoR I. The plasmid DNA and cytochrome c DNA fragments were ligated with DNA ligase (Toyobo) and the resulting recombinant plasmid, pLitmus-cytc, was transformed into *E. coli* DH5α strain. The pLitmus-cytc plasmid was identified with BamH I and EcoR I digestion and DNA sequence analysis. The plasmid containing GFP gene fragment, pLitmus-GFP, was constructed as described similarly for negative control.

### DsRNA synthesis and RNAi

The pLitmus-cyt c plasmids were extracted using QIAGEN spin miniprep kit (QIAGEN GmbH, Hilden, Germany) and linearized with BamH I and EcoR I digestion. Then the target DNA fragment was purified and used as a template to produce dsRNA. RNA was synthesized using a T7 transcription kit. The dsRNA products were ethanol precipitated and suspended in water and quantified. 5 µg of dsRNA was visualized by 1.5% agarose gel. The dsRNA was stored at −80°C. For *Spodoptera litura* cytochrome c dsRNA mediated RNA inference assay, 5×10^5^ cells/ml were seeded into 24-well plates and cultured in serum-free medium for 2 hr. Cells in each well were transfected with a mixture of 1 µg dsRNA, 5 µl Cellfectin reagent (Invitrogen, USA) and 25 µl serum-free medium according to manufacture's protocol. After cells were incubated for 6 h at 28°C, serum-free medium were replaced with medium supplemented with 10% FBS. Cells were grown at various time intervals and then infected with AfMNPV at MOI of 5. After virus inoculum was removed at 2 h post infection, cells in new medium were cultured for 10 hr. Cells were transfected with pFBDC plasmid and GFP dsRNA as a negative control. Cytochrome c mRNA expression in Sl-1 cells was measured with semi-quantitative RT-PCR with β-actin gene used as an internal control. Briefly, the cells were infected with AfMNPV after the cells had been treated with dsRNA for 24, 48 and 72 h. Then the total RNA in each treatment was isolated at 10 h post infection. cDNAs were synthesized by reverse transcription and a forward primer (5-TGCCACACTGTTGAAGCCGGT-3) and a reverse primer(5-GATCTGCACGCTCGTTTGCCT-3) for cytochrome c gene were used together to produce PCR products. The PCR products were electrophorised on 1.5% gel and analyzed with Bio-Rad software Quantity One.

To further confirm the effect of RNAi of cytochrome c in Sl-1 cells, the abundance of cytochrome c was demonstrated by western-blot at the different time points after the transfection of cytochrome c dsRNA. The protocol was briefly described as follow. The cells were harvested at 0, 24, 48, and 72 h after the transfection as described above. The concentration of total protein was determined according to the method of BCA after addition of lysis buffer containing protease inhibitors and centrifugation. Total cellular proteins were separated by 12% SDS-PAGE and transferred onto nitrocellulose membranes. The membrane was blocked with TBS containing 5% skimmed milk for 1 h at room temperature and then incubated for 1 h with the monoclonal anti-β-tubulin (Sigma-Aldrich, St. Louis, MO, USA) and anti-cytochrome c mouse antibody (BD Biosciences) as primary antibody at a 1∶1000 dilution. Finally, the membrane was washed with blocking buffer and then incubated with peroxidase-conjugated goat anti-mouse immunoglobulin G (Promoter biotechnology, China) as secondary antibody for 1 h at room temperature. The specific bands were visualized by revelation with 3-amino-9-ethylcarbazole (AEC).

### Assay of caspase activity

To assay caspase activity in Sl-1 cells interfered with dsRNA, after cells were transfected with dsRNA at different times, the cells had been infected with AfMNPV at MOI of 5 for 10 h. The cells were collected by centrifugation at 13,500×g for 5 min at 4°C. The cells were suspended in cell lysis buffer and incubated on ice for 10 min and the cell lysates were centrifuged at 13,500×g for 20 min at 4°C. The resultant supernatant was collected and quantified by Biophotometer (Eppendorf, Germany). Caspase 3 and caspase 9 activities were determined by measuring the proteolytic cleavage of the fluorogenic substrate Ac-DEVD-AFC and Ac-LEHD–AFC (BD Sciences), respectively. The reaction mixtures consisted of 25 µg of extracts and 100 mM substrate in 250 µl assay buffer (50 mM Hepes-KOH, pH 7.5, 75 mM NaCl, 1 mM EDTA-Na_2_, 2 mM DTT, 0.5% Chaps,10% sucrose) were incubated at 37°C for 1 h. The reaction was terminated with a dilution of 1 ml ice-cold assay buffer (pH7.5), and fluorescence was measured using a spectrofluorimetry at an excitation wavelength of 400 nm and an emission wavelength of 505 nm. Caspase 3 activities were expressed in relative fluorescent units per fraction. For assaying caspase-3 activation in the cell-free apoptotic system induced by cytochrome c, the reactive mixtures containing cell-free extracts, cytochrome c was incubated at 28°C for various time, each reactive mixture was centrifuged at 13,500×g for 10 min. After removal of pellets, the supernatants were used as caspase 3 activation assay. Caspase 3 activities were determined and analyzed as mentioned above.

Z-LEHD-FMK was a specific inhibitor of caspase-9. In order to determine the relationship between caspase 9 and caspase 3 cascade, Sl-1 cells were infected by AfMNPV at moi = 5 in the precence or absence of caspase 9 inhibitor at different final concentrations (20, 40 and 60 µM, respectively) and caspase 3 activities were measured by a spectrofluoremeter at 10 h post-infection according to the method described as above.

### Flow cytometric analysis of apoptotic cells

The Annexin V-FITC kit was used for flow cytometric analysis of apoptosis in Sl-1 cells. Briefly, after cells were transfected with dsRNA at different time points, the cells were inoculate with AfMNPV for 10 h. The cells were pelleted by centrifugation at 800×g for 10 min and pellets were washed twice with PBS, and then resuspended with prediluted binding buffer. The concentration of cells was adjusted to 1×10^6^ cells/ml, 5 µl Annexin V-FITC was added to 195 µl cell supernatant and gently mixed, incubated at room temperature for 10 min then stained with 10 µl of 20 µg/ml propidium iodide for 20 min.The cells were examined and analyzed by flow cytometry on 488 nm fluorescence/area histogram.

### Treatment of AfMNPV-induced Sl-1 cells with FSBA

5×10^5^ cells/ml were seeded into flasks or 6-well plates and grown over night. Then the medium was replaced with the fresh medium containing10% FBS and FSBA at different concentrations (0, 50 and 100 µM). After 3 h of the pretreatment of the cells with FSBA, the cells were washed twice with fresh medium and inoculated with AfMNPV at multiplicity of infection (MOI) of 5 for 3 hr. The virus inoculum was removed and cells washed twice with serum-free medium and then cultured in Grace's medium supplemented with 10% FBS and FSBA at different concentrations. the relative caspase-3 activity and the percentage of apoptotic cells were measured at 12 h post-infection according to the methods described as above.
